# Candidate Spatial Niches Associated with Gastric Cancer Metastasis and Treatment Resistance: Functional Classification, Evidence Boundaries, and a Translational Framework

**DOI:** 10.3390/cells15181676

**Published:** 2026-09-16

**Authors:** Yi Huang, Jingcheng Zhang, Jiaheng Lou, Bo Bian, Mingsi Zhang, Taowei Gou, Tao Jiang, Guangji Zhang

**Affiliations:** 1 School of Basic Medical Sciences, Zhejiang Chinese Medical University, Hangzhou 310053, China; zjhy7493@163.com (Y.H.); 15328236888@163.com (J.Z.); ljh990704@163.com (J.L.); bianbobianbo@163.com (B.B.); 17757197011@163.com (M.Z.); goutaowei11@163.com (T.G.); 2 Zhejiang Key Laboratory of Blood-Stasis-Toxin Syndrome, Zhejiang Chinese Medical University, Hangzhou 310053, China; 3 Traditional Chinese Medicine “Preventing Disease” Wisdom Health Project Research Center of Zhejiang, Hangzhou 310053, China

**Keywords:** gastric cancer, metastasis, treatment resistance, candidate spatial niche, evidence boundaries, tumor microenvironment, clinical translation

## Abstract

**Highlights:**

**What are the main findings?**

- A four-stage evidence framework and a minimum criteria chain are proposed to distinguish descriptive spatial patterns from candidate functional spatial niches in gastric cancer.
- Five spatial framework categories are organized according to dominant spatial contexts and malignant functional outputs, with explicit evidence boundaries defined for metastasis and treatment resistance.

**What are the implications of the main findings?**

- Spatial-niche interpretation provides a structured approach for integrating spatial omics, multiplex imaging, pathology, and mechanistic evidence while maintaining appropriate evidence boundaries.
- Quantifiable niche metrics may support future risk stratification, treatment-response prediction, and spatially guided functional validation and combination strategies.

**Abstract:**

Gastric cancer metastasis and poor treatment responses are spatially heterogeneous; however, single-marker positivity, cellular enrichment, or simple colocalization alone is insufficient to establish a functionally meaningful spatial niche. Within this framework, “treatment resistance” encompasses primary nonresponse, reduced treatment sensitivity, insufficient local drug delivery, post-treatment residual survival, acquired resistance, and interlesional differences in therapeutic efficacy. We propose a minimum criteria chain for candidate spatial niches comprising four elements: a specified spatial context; neighborhoods characterized using prespecified and transparently reported spatial rules; local functional programs concordant with these neighborhoods; and associations with malignant phenotypes or clinical outcomes. Based on the principle of linking dominant spatial contexts with their predominant malignant functional outputs, we summarize four candidate niches within primary tumors—the invasive front–tumor–stroma interface, immune exclusion–immune evasion, hypoxia–metabolic adaptation, and perivascular supportive niches—as well as an organ-context-dependent adaptive niche-forming process at metastatic sites. The level of evidence supporting these categories varies substantially. Immune-exclusion-related spatial states currently have relatively stronger links to treatment-response cohorts; however, rigorous gastric-cancer-specific in situ validation remains limited, and no candidate niche has yet achieved full clinical validation. Invasive-front and hypoxic niches are supported primarily by pathological or mechanistic evidence, whereas evidence for perivascular niches remains limited despite emerging longitudinal treatment-response observations, and metastatic niches outside the peritoneum require stronger longitudinal, local functional, and spatial-perturbation validation. This framework aims to standardize spatial-niche terminology and evidence interpretation and guide future quantification, validation, and potential clinical translation in gastric cancer.

## 1. Introduction: From Spatial Heterogeneity to a Spatial-Niche Framework

Adverse outcomes in patients with gastric cancer extend beyond local progression of the primary tumor and include postoperative recurrence, distant metastasis, and failure of systemic therapy [1,2]. Multicenter studies have demonstrated that substantial recurrence risk persists even after curative-intent resection: a recurrence rate of 29.9% was reported in a United States multi-institutional cohort [3], whereas a Chinese multicenter cohort reported a rate of 60.8% [1], with distant metastasis representing an important pattern of recurrence [4]. Although systemic therapies, including chemotherapy, HER2-targeted therapies, and immune checkpoint inhibitors, have improved outcomes in selected patients with advanced gastric cancer, the prognosis of unresectable, recurrent, or metastatic disease remains poor [5,6]. Therefore, metastasis and treatment resistance remain major challenges in both gastric cancer research and clinical management.

Current clinical management relies on an integrated diagnostic and therapeutic pathway. Cross-sectional imaging and endoscopy with biopsy are used to determine disease extent and establish histopathological diagnosis, whereas biopsy specimens enable treatment-relevant pathological and molecular characterization, including assessment of HER2, MSI/MMR, PD-L1 CPS, and, where appropriate, CLDN18.2. In selected patients with locally advanced disease, staging laparoscopy can detect otherwise occult peritoneal dissemination and facilitate multidisciplinary treatment planning [7]. Molecular characteristics can also influence responses to neoadjuvant or conversion therapy. Recent clinical studies evaluating HER2 and MSI status highlight the importance of integrating pretreatment tissue profiling with treatment response and survival outcomes [8]. For resectable disease, guideline-concordant gastrectomy with appropriate lymphadenectomy remains the cornerstone of treatment, and indocyanine-green fluorescence imaging may facilitate lymphatic mapping and lymph-node retrieval during laparoscopic surgery [9]. D2 lymphadenectomy remains the reference procedure for most cN-positive or cT2–4 resectable tumors; more extensive D2-plus approaches are not routine substitutes for D2, but may be considered for selected patterns of nodal risk, after systemic therapy, or in specialized centers according to contemporary guidance and multidisciplinary judgment [10]. After curative-intent treatment, follow-up incorporating clinical assessment, endoscopy, laboratory testing, and imaging should be individualized according to pathological stage, recurrence risk, symptoms, prior treatments, and available salvage options [10]. These clinical steps determine which tissue compartments are sampled, which biomarkers are available for comparison, and which treatment-specific outcomes a spatial metric must improve upon.

The tumor microenvironment is a key determinant of local invasion, metastasis, immune evasion, and treatment resistance in gastric cancer [11,12]. Its influence depends not only on the presence of specific cell types or molecules but also on their spatial organization within local tissue ecosystems [13,14]. Conventional component-oriented studies have primarily focused on individual cellular or molecular components, including tumor cells, cancer-associated fibroblasts (CAFs), tumor-associated macrophages (TAMs), T cells, abnormal vasculature, and metabolic programs [15,16]. Such studies are valuable for identifying key molecules and cellular states, but alone they are insufficient to explain why complex malignant phenotypes persist in specific regions or why the same components are associated with different metastatic tendencies or treatment responses across distinct tissue contexts.

Emerging technologies, including spatial transcriptomics, multiplex in situ imaging, spatial proteomics, spatial metabolomics, and digital pathology, now enable integrated analysis of cellular composition, spatial relationships, and local functional programs while preserving tissue architecture [17,18,19,20,21]. These platforms differ substantially in spatial resolution, molecular throughput, cell-identification capacity, and their ability to support functional interpretation. Therefore, the availability of spatial information alone does not indicate that a functionally meaningful spatial niche has been identified.

### 1.1. Conceptual Boundaries and Minimum Criteria for Candidate Spatial Niches

In this review, spatial patterns, spatial domains, and cellular neighborhoods are not considered a strictly linear hierarchy. Spatial patterns describe nonuniform distributions of markers, cell types, or pathological features. Spatial domains refer to regions defined by morphology, expression profiles, or tissue architecture. Cellular neighborhoods describe local cellular composition and cell–cell or cell–structure relationships according to explicit spatial rules. These forms of evidence provide complementary descriptions of local tissue organization; however, none of them are automatically equivalent to a functionally defined niche. We categorize evidence supporting candidate niches into four stages: (1) descriptive spatial evidence; (2) candidate-niche evidence; (3) functional-support evidence; and (4) clinical-validation support. The minimum criteria chain for a candidate spatial niche comprises four elements: (1) a clear tissue location or spatial context; (2) a cell–cell or cell–structure neighborhood characterized using a prespecified and transparently reported spatial rule; (3) a concordant local functional program; and (4) an association with malignant phenotypes or clinical outcomes, such as metastasis, post-treatment residual disease, or a specific treatment response [22,23,24,25,26,27,28].

Positivity for a single marker, cellular enrichment, isolated colocalization, or a purely prognostic association may indicate spatial heterogeneity or identify potentially relevant regions; however, none of these observations alone are sufficient to establish a candidate spatial niche. Advancement to functional-support evidence requires spatial perturbation, spatially preserved models, or longitudinal evidence. Clinical-validation support additionally requires independent cohort replication and, where appropriate, cross-platform replication, together with evidence supporting incremental clinical value. Accordingly, we evaluate the five framework categories across three evidence dimensions: (1) in situ spatial evidence from gastric cancer; (2) functional or mechanistic evidence; and (3) clinical outcome or treatment-response evidence. For each dimension, the evidence source is specified as gastric-cancer evidence or cross-cancer support.

### 1.2. Classification Principles for the Five Framework Categories and Boundaries of the Framework

This review does not aim to provide an exhaustive catalog of all spatial niches in gastric cancer; rather, it focuses on candidate spatial niches associated with adverse malignant phenotypes, including metastasis and treatment resistance. Spatial states associated with effective antitumor immunity, treatment sensitivity, or favorable clinical outcomes are outside the primary scope of this classification; however, they remain important comparators when continuous spatial variables are calibrated. Therefore, tertiary lymphoid structures (TLSs) and lymphocyte-rich intratumoral regions are considered favorable spatial reference states rather than an additional adverse niche category. Within this scope, the five framework categories are organized according to the principle of linking dominant spatial contexts with their predominant malignant functional outputs, rather than being defined by a single anatomical structure or cell type. The first four categories are located mainly within primary tumors. The invasive front–tumor–stroma interface niche is characterized primarily by disruption of tissue interfaces and local infiltrative expansion, the immune exclusion–immune evasion niche by failure of immune clearance, the hypoxia–metabolic adaptation niche by stress adaptation and survival support under low-perfusion conditions, and the perivascular supportive niche by spatial proximity at the tumor–vascular interface accompanied by chemotactic, adhesive, and vascular-remodeling programs. The fifth category occurs at metastatic sites and describes an organ-context-dependent adaptive process that emerges after gastric cancer cells colonize different organs.

The five categories were included because they recur in gastric cancer studies, have identifiable tissue contexts and at least one quantifiable neighborhood relationship, and show associations with metastatic processes or specific treatment-failure phenotypes. Importantly, individual categories are not required to demonstrate associations with both metastasis and all treatment modalities simultaneously. We prioritize in situ spatial evidence from gastric cancer and use nonspatial mechanistic studies from gastric cancer and cross-cancer studies only as supplementary support for functional interpretation. The perivascular category is retained despite its relatively lower evidence maturity because it represents a directly identifiable tumor–vascular spatial unit within gastric cancer tissue. By contrast, the premetastatic niche is established before disseminated tumor cells arrive and differs from the present framework in both temporal context and the unit of assessment. Its exclusion is therefore based on both conceptual considerations and differences in available evidence. A treatment-remodeled residual-survival niche is not listed as a separate category because it is more plausibly a shared consequence of hypoxic, immune-excluded, and vascular-related states following treatment selection or remodeling, while paired pre- and post-treatment spatial evidence in gastric cancer remains limited.

Classification is performed according to a prespecified adjudication sequence. First, the tissue unit is assigned to the primary-tumor or metastatic-site setting. Second, the dominant anatomical/spatial anchor is identified: the deepest invasive margin supports an invasive-front assignment; the tumor-nest–stroma immune-access boundary supports an immune-exclusion assignment; a viable, poorly perfused, vessel-distant, or necrotic-border region supports a hypoxia–metabolic assignment; and a direct tumor–vascular interface supports a perivascular assignment. Third, the principal malignant functional output is required to be concordant with that anchor. Coexisting CAF, ECM, myeloid, hypoxic, vascular, and immune features are recorded as secondary annotations rather than automatically generating multiple primary labels. If no dominant anchor and output can be prespecified, the region should be reported as mixed or unresolved. At metastatic sites, peritoneal metastasis is currently the best-supported gastric-cancer-specific example; lymph-node, brain, and other organ sites should be analyzed as distinct candidate subtypes rather than pooled into a uniform metastatic niche.

Within this review, “treatment resistance” is used as an overarching concept to describe adverse treatment responses that are present before therapy or emerge during treatment and are related to the tumor and its local tissue environment. These include primary nonresponse or limited benefit from immunotherapy, reduced sensitivity to chemotherapy or targeted therapy, insufficient local drug delivery, survival of residual cells after treatment, acquired resistance, and differences in therapeutic efficacy between primary and metastatic lesions. Dynamic terms such as “acquired resistance,” “treatment-induced formation,” or “continuous evolution” are used only when paired pre- and post-treatment or longitudinal evidence is available; cross-sectional spatial differences are used only to support pre-existing heterogeneity, interlesional state differences, or a reasonable inference of treatment-selective retention.

### 1.3. Literature Search and Rules for Evidence Interpretation

This article is a narrative review with a conceptually integrative framework. Literature identification was performed through searches of PubMed and Web of Science from database inception to 13 August 2026, while Google Scholar was used only for supplementary citation chasing and identification of additional references. Search concepts covered gastric cancer, metastasis, recurrence, treatment nonresponse, reduced treatment sensitivity, acquired resistance, residual survival, spatial niche or domain, cellular neighborhood, spatial transcriptomics, multiplex in situ imaging, digital pathology, invasive front, immune exclusion, hypoxia, metabolic adaptation, perivascular context, metastatic-site adaptation, and peritoneal dissemination, alone and in combination. Eligible evidence included original human gastric-cancer studies that localized a feature in tissue, quantified a spatial relationship, tested a local functional mechanism, or linked a spatial variable to a malignant phenotype or treatment outcome. Cross-cancer and nonspatial studies were retained only when they provided clearly labeled mechanistic, methodological, or clinical-validation precedent support. Dissociation-based datasets without tissue-location information were not classified as direct in situ niche evidence, although they could contribute complementary support.

Screening decisions and evidence-stage assignments were reviewed by the authors, and disagreements regarding study eligibility or evidence categorization were resolved through discussion and consensus.

To examine framework usability, two authors independently applied the classification framework to representative studies from the evidence map, with the corresponding two-rater assessment results provided in Supplementary Table S4. Agreement was assessed for both primary framework assignment and evidence-stage assignment. Complete concordance was observed for both assessments (8/8 cases, 100%). Given the small illustrative sample and the involvement of both raters in framework development, this exercise was not interpreted as a formal estimate of inter-rater reliability and may be subject to familiarity-related bias. Instead, this observed agreement was used only to illustrate the practical consistency of framework application rather than to establish reproducibility or validation. Future independent applications should incorporate predefined procedures for resolving discrepancies, such as discussion and consensus.

Because the original historical database exports and screening counts were not retained, a formal rerun was performed during revision using locked PubMed and Web of Science Core Collection search strings while preserving the original publication cutoff of 13 August 2026; Google Scholar was used only for supplementary citation chasing and was not included in the formal denominator. Across seven PubMed and seven Web of Science search sets, the rerun yielded 5388 gross records (PubMed n = 2280; Web of Science n = 3108). After transitive deduplication by DOI, PMID, and normalized exact title, 2693 unique records remained and were subjected to record-level relevance screening. The final bounded evidence map contains 66 entries: 51 captured by the formal rerun and 15 retained through supplementary citation chasing or as explicitly labeled complementary/validation-precedent sources. These two splits represent different dimensions of evidence organization: the 51/15 split describes the retrieval pathway through which studies entered the evidence map, whereas the 49/17 split describes the subsequent evidence-stage classification according to the role of each study within the framework. Of these, 49 are staged gastric-cancer spatial/comparator studies (Stage 1, n = 17; Stage 2, n = 23; Stage 3, n = 9; Stage 4, n = 0) and 17 are complementary sources outside the gastric-cancer niche evidence-stage hierarchy (N/A). Because this review is not presented as a systematic review and a historical full-text exclusion log was not retained, no retrospective PRISMA-style full-text denominator or exclusion-reason distribution was reconstructed. Supplementary Table S1 reports the study-level design, cohort/sample size or analytic units, platform, sampling and treatment context, endpoint, primary framework assignment, secondary annotations/overlap, evidence source, retrieval route, evidence stage, and principal evidence boundary for each mapped entry. The associated Search Log reports the locked search strings, database counts, eligibility boundaries, deduplication audit, screening transparency, and study-level retrieval provenance. Evidence stages were assigned using the criteria in Table 1; when several evidence types were present, the highest stage directly supported by the study design was recorded without promoting cross-cancer support to gastric-cancer validation.

Longitudinal treatment-response studies providing paired or serial spatial analyses were assigned to the functional-support stage when the study design directly supported treatment-associated spatial remodeling or temporal functional change.

## 2. Functional Classification of Candidate Spatial Niches Associated with Gastric Cancer Metastasis and Treatment Resistance

Multimodal spatial studies have revealed substantial spatial heterogeneity in local invasion, immune-clearance failure, hypoxic adaptation, and vascular-associated spatial organization and signaling programs, as well as metastatic-site reorganization in gastric cancer. The five spatial framework categories are compared below according to the classification principles described above [29,30]. These categories should not be interpreted as discrete, mutually exclusive regions with sharply defined boundaries. A single local tissue unit may simultaneously display CAF enrichment, extracellular matrix remodeling, myeloid-cell infiltration, hypoxia, and immune exclusion [31,32]. Primary classification should therefore follow the anatomical-anchor and functional-output adjudication sequence described in Section 1.2, whereas overlapping programs should be retained as secondary annotations. For example, an invasive-front region exhibiting hypoxia and CD8^+^ T-cell exclusion should remain primarily classified according to its deepest-margin interface and infiltrative output. Conversely, a region lacking a prespecified dominant anchor should be reported as mixed or unresolved rather than forced into a single category.

**Figure 1:**
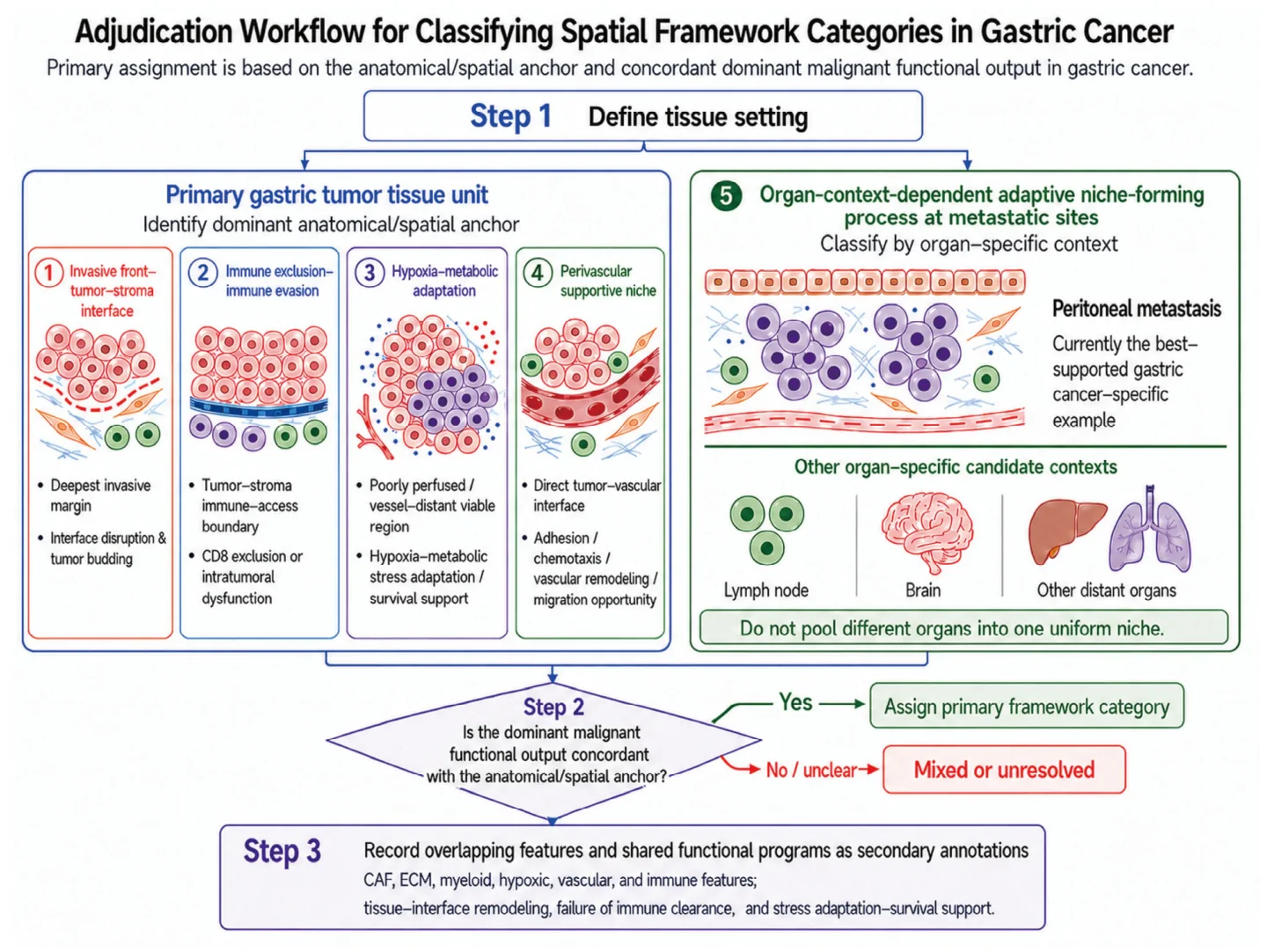
Classification and adjudication framework for spatial framework categories in gastric cancer. Framework categories are assigned using a prespecified sequence integrating tissue setting, dominant anatomical/spatial anchors, and concordant malignant functional outputs. Primary classification is determined by the spatial context that most plausibly explains the dominant malignant process, whereas overlapping stromal, immune, metabolic, and vascular features are retained as secondary annotations rather than generating multiple primary labels. Regions lacking a prespecified dominant spatial anchor and functional output should be reported as mixed or unresolved. Metastatic contexts, including peritoneal, lymph-node, brain, and other organ sites, are evaluated separately according to their organ-specific spatial context rather than being pooled into a uniform metastatic-site niche. This framework provides a structure for evidence interpretation and does not imply established causal relationships or clinical validation. Abbreviations: CAF, cancer-associated fibroblast; ECM, extracellular matrix.

The framework shown in Figure 1 establishes the classification sequence used throughout this review. Candidate spatial niches are first identified according to their tissue setting and dominant anatomical/spatial anchor, followed by evaluation of whether the associated malignant functional output is concordant with that spatial context. Coexisting stromal, immune, metabolic, and vascular features are retained as secondary annotations rather than used to generate multiple primary classifications. According to the criteria chain and evidence interpretation rules summarized in Table 1, the five spatial framework categories are subsequently compared across five dimensions: spatial context, neighborhood composition, functional programs, malignant outputs, and the current evidence base.

**Figure 2:**
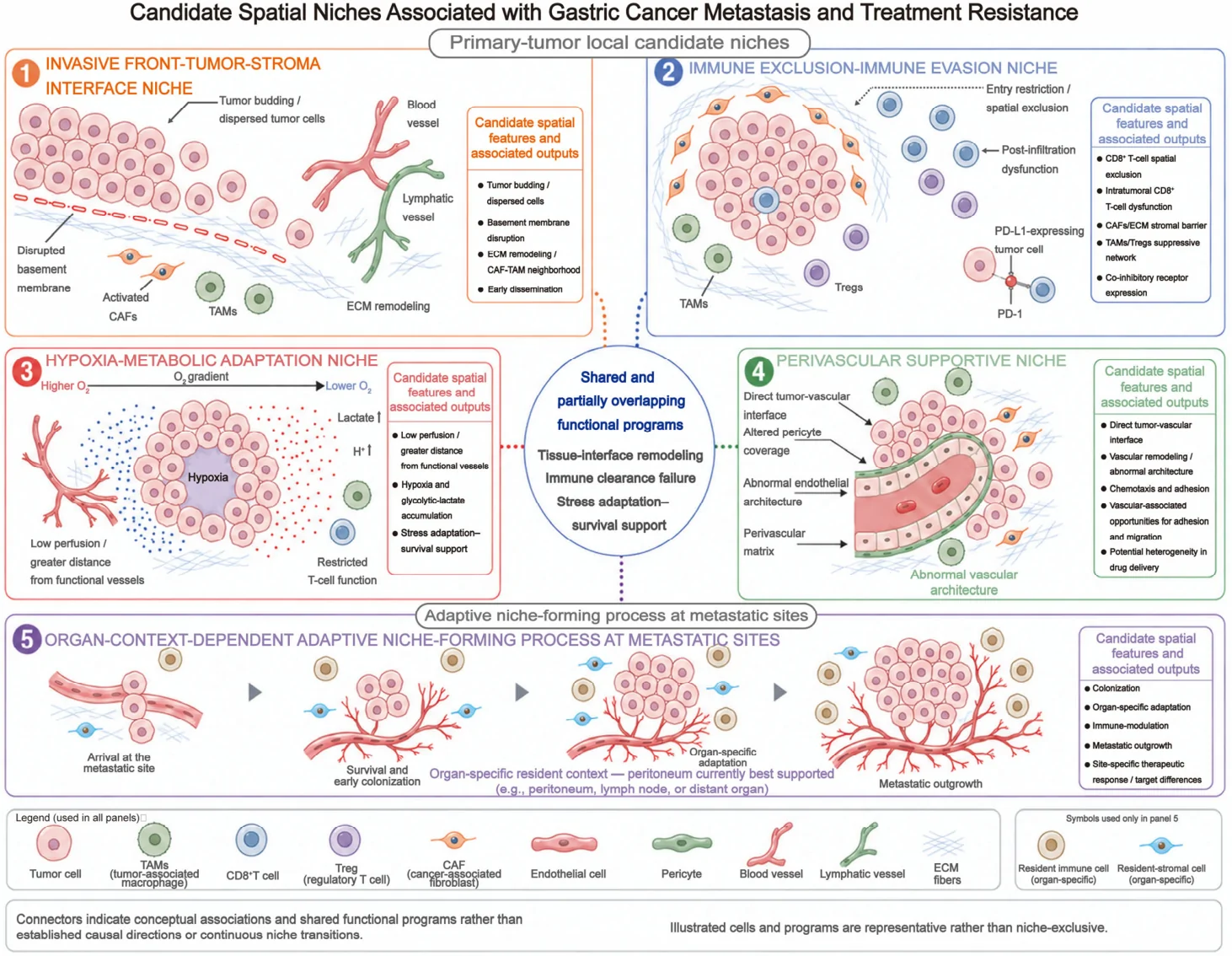
Schematic representation of candidate spatial niches associated with gastric cancer metastasis and treatment resistance and of cross-niche functional programs. The first four categories represent local candidate niches within the primary tumor, whereas the fifth represents an organ-context-dependent adaptive niche-forming process at metastatic sites. Tissue-interface remodeling, failure of immune clearance, and stress adaptation–survival support, shown in the central region, are shared functional programs used for cross-niche comparison: the same program may generate different outputs in different spatial contexts, including local infiltrative expansion, immune evasion, residual survival, or distant colonization. The connecting lines indicate only conceptual associations and potential program overlap and do not represent established unidirectional causal relationships or continuous niche evolution. The cells and molecules shown are representative features rather than strictly niche-specific markers. Abbreviations: CAF, cancer-associated fibroblast; ECM, extracellular matrix; TAMs, tumor-associated macrophages; Treg, regulatory T cell; PD-1, programmed cell death protein 1; PD-L1, programmed death-ligand 1.

The conceptual relationships among the five framework categories and shared functional programs are illustrated in Figure 2.

An illustrative two-rater application of the framework, including primary framework assignment and evidence-stage agreement, is provided in Supplementary Table S4.

### 2.1. Invasive Front–Tumor–Stroma Interface Niche

The invasive front–tumor–stroma interface niche is located at the deepest infiltrative margin of the primary gastric tumor, where tumor nests interface with the surrounding stroma. Rather than being defined solely by the presence of migratory tumor cells, this niche is characterized by the spatial co-occurrence of tumor budding or dispersed cancer cells, reduced basement-membrane continuity, collagen and extracellular matrix remodeling, and supportive stromal or myeloid-cell neighborhoods within the same tissue interface [33,34,35,36,37,38,39]. This histological context is primarily associated with the disruption of tissue barriers within the primary tumor, stromal infiltration by tumor cells, and features consistent with early metastatic dissemination.

An operational identification strategy may begin with characterization of the pathological interface: Hematoxylin and eosin (H&E) staining and Pan-CK may help identify the deepest invasive front and tumor budding; collagen IV may assess basement-membrane continuity; and FAP and CD68 may characterize fibroblast and macrophage-associated neighborhoods, whereas LAMC2, MMP7, MMP14, and related markers can supplement the assessment of interface-remodeling programs [33,40,41,42,43,44,45,46,47,48,49,50,51].

Taken together, current evidence supporting the invasive-front niche primarily consists of pathological localization of tumor budding and invasive-front markers, clinical associations with lymph-node metastasis, recurrence, and survival, as well as selected studies describing the spatial distribution of CAFs and TAMs [33,45,51,52,53,54]. Whether stable co-occurrence of FAP^+^ CAFs, SPP1^+^ or CD163^+^ TAMs, and tumor buds within the same local neighborhood is functionally required for interface disruption and early dissemination remains unresolved, owing to the lack of cross-cohort replication, spatial perturbation studies, and functional models preserving tissue architecture. The principal biological outputs associated with this niche include local infiltrative expansion, lymphovascular invasion, and early dissemination. Current treatment-related evidence is more appropriately interpreted as supporting this niche as a histopathological feature associated with increased perioperative metastatic or recurrence risk, whereas direct evidence connecting this niche to resistance to specific chemotherapy, targeted therapies, or immunotherapies remains limited [44,45,55,56,57,58].

### 2.2. Immune Exclusion–Immune Evasion Niche

The immune exclusion–immune evasion niche encompasses two related but distinct spatial states that should be evaluated separately. The first is “entry restriction/spatial exclusion,” in which effector T cells remain concentrated at the tumor margin or surrounding stroma and show limited access to tumor nests. The second is “post-infiltration functional dysfunction,” in which T cells have entered tumor regions but exhibit reduced cytotoxic programs, limited clonal expansion, or suppression by local inhibitory networks. Both states may contribute to impaired immune clearance, but they differ in spatial organization, analytical metrics, and potential therapeutic implications [59,60,61,62,63].

#### 2.2.1. Entry Restriction and Spatial Exclusion

Entry restriction should primarily be evaluated based on the spatial relationship between tumor-nest boundaries and effector T-cell localization. Pan-CK or EPCAM can delineate tumor regions, whereas spatial exclusion may be quantified using measures such as the ratio of CD8^+^ T-cell density within tumor nests versus the stroma, distance between CD8^+^ T cells and the nearest tumor cell, tumor–CD8 adjacency defined by a prespecified and transparently reported spatial rule, and CAF/ECM barrier thickness [61,64]. FAP, collagen, or fibronectin can characterize the stromal barrier, whereas CD68, FOXP3, and local myeloid/Treg neighborhoods can provide complementary information on the immunosuppressive context [14,45,65,66]. These measurements should be interpreted as spatial variables requiring prespecified analytical rules and appropriate validation strategies. Detailed considerations regarding continuous versus dichotomized spatial metrics, threshold selection, robustness assessment, and reproducibility evaluation are discussed in Section 3.4.

#### 2.2.2. Post-Infiltration Functional Dysfunction

Post-infiltration functional dysfunction should be evaluated only after confirming intratumoral T-cell localization and should incorporate cytotoxic/effector programs such as GZMB, PRF1, and IFNG, information on proliferation or clonal expansion, and co-inhibitory receptors including PD-1, TIM-3, LAG-3, TIGIT, and CTLA-4. Expression of co-inhibitory receptors may indicate activation-associated inhibition or an exhaustion-related state; however, it does not by itself demonstrate loss of cytotoxic function.

In gastric cancer, spatial colocalization and functional associations between CCL2^+^ CAFs and STAT3-activated macrophages have been supported by spatial transcriptomics, in vitro recruitment and activation assays, and mouse models [14]. The association between SPP1^+^ TAMs and exhausted-like CD8^+^ T cells is derived primarily from single-cell ligand–receptor analyses, integration with bulk datasets, and animal models. It should therefore be interpreted as a putative intercellular communication axis rather than as an in situ spatial interaction that has been directly demonstrated by imaging [62]. In addition, mechanistic studies showing that TAM-derived CXCL8 can induce immunosuppressive phenotypes provide complementary nonspatial functional support for myeloid-mediated immune evasion [67].

From a therapeutic perspective, spatial transcriptomic, multiplex-imaging, and treatment-cohort studies have associated macrophage enrichment, CD8^+^ T-cell states, and specific spatial organizational features with primary nonresponse, limited clinical benefit, or a lower pretreatment probability of response to immune checkpoint inhibitors or combined immunotherapy and antiangiogenic therapy [61,65,68]. Entry restriction is more directly suited to analyses of stromal barriers and chemotactic regulation, whereas post-infiltration functional dysfunction requires greater emphasis on effector programs and on myeloid-, Treg-, and checkpoint-mediated suppression. Whether these spatial neighborhoods are functionally required for treatment nonresponse remains unresolved and will require direct perturbational or longitudinal validation.

Favorable spatial states remain important reference comparators for calibration, although they are not included among the five adverse framework categories. Studies of TLS in gastric cancer have shown that TLS location, cellular composition, and intercellular proximity may be associated with organized antitumor immune activity and more favorable immunotherapy-related outcomes [69]. TLS are therefore considered a favorable spatial reference state against which immune exclusion and post-infiltration dysfunction can be interpreted. They are not designated as a sixth principal niche because the prespecified scope of this review focuses specifically on spatial states associated with metastasis, treatment failure, residual survival, or other adverse malignant phenotypes.

### 2.3. Hypoxia–Metabolic Adaptation Niche

The hypoxia–metabolic adaptation niche primarily refers to viable tumor regions that are located distant from effectively perfused vessels, exposed to impaired perfusion caused by abnormal vasculature, or positioned adjacent to necrotic borders. The defining feature of this candidate niche is the spatial co-occurrence of a low-perfusion context with hypoxia–metabolic adaptation programs, rather than necrosis itself or positivity for an individual HIF-related marker [70,71,72,73,74,75,76]. These regions may exhibit enhanced glycolysis, lactate accumulation, extracellular acidification, redox-homeostasis adaptation, and stress-survival programs, and they may also be associated with suppression of local immune effector function.

Candidate designation should integrate spatial features such as vessel distance and proximity to necrosis with evidence of metabolic adaptation programs. CD31 can localize vascular structures but cannot establish functional perfusion. Where feasible, functional perfusion measurements, hypoxia probes, or imaging-based indicators should therefore be incorporated. CAIX may reflect relatively persistent hypoxia and acid–base adaptation, whereas GLUT1, LDHA/LDH-5, and MCT4 may provide information regarding glucose uptake, lactate production, and lactate export, respectively [72,77,78,79,80,81,82]. The concurrent presence of these programs in viable tumor cells distant from vessels or adjacent to necrotic regions can support a candidate niche. Current evidence in gastric cancer is derived largely from IHC, pathological correlations, and nonspatial metabolic-mechanism studies, whereas studies integrating poor perfusion, intercellular metabolic division of labor, and immune dysfunction within the same in situ neighborhood remain insufficient. These markers should also be interpreted in relation to cellular source and spatial location; expression of LDHA or MCT4 alone cannot substitute for direct measurements of lactate concentration, metabolic flux, or intercellular lactate transport.

The principal metastasis-related outputs associated with this niche include stress tolerance, anoikis resistance, and survival adaptation during dissemination [74,83,84,85,86]. Gastric cancer studies have reported associations of HIF-related hypoxic programs with reduced chemosensitivity, chemoresistant phenotypes, and treatment outcomes [87,88,89,90], whereas insufficient drug exposure caused by poor perfusion is supported mainly by tumor drug-delivery studies and cross-cancer mechanistic evidence [91]. These findings support an association between hypoxia/metabolic stress and reduced treatment sensitivity or residual survival. However, simultaneous measurement of perfusion, metabolic state, drug exposure, and post-treatment residual cells within the same spatial region remains necessary.

### 2.4. Perivascular Supportive Niche

In this review, the perivascular supportive niche is operationally defined as a “candidate spatial unit at the tumor–vascular interface,” in which gastric cancer cells exhibit quantifiable spatial proximity to vascular endothelial cells, pericytes, and the perivascular matrix together with local chemotactic, adhesive, or vascular-remodeling programs [92,93,94,95,96,97,98]. The core criterion is not merely tumor-cell proximity to vessels, but a clearly identifiable and transparently characterized vascular-interface structure accompanied by related molecular or cellular programs. This definition does not imply that such regions have already been demonstrated to promote intravasation or function as post-treatment sanctuaries.

Pan-CK can be used to localize tumor cells, whereas CD31/ERG can delineate vascular endothelial structures. RGS5, CSPG4, PDGFRβ, α-SMA, and desmin should be interpreted in the context of their perivascular localization when assessing pericyte-associated features, whereas CXCL12, VEGFA, integrins, selectins, and VCAM-1 should be considered complementary indicators of chemotactic, adhesive, or remodeling programs only when their cellular sources and spatial locations are clearly defined [99,100,101,102,103,104,105,106,107,108]. Studies in gastric cancer have reported spatial proximity between tumor cells and vascular structures, involvement of vascular-associated signaling in migration or adhesion, and associations between abnormal vasculature and local tissue states. Evidence related to lymphatic proximity, lymphangiogenesis, and lymphatic invasion reflects the tumor–lymphatic interface, which should not be considered structurally equivalent to perivascular support; here, such evidence is treated as complementary support for the invasive front and lymphovascular invasion rather than as a core criterion for the perivascular niche [38,39]. These vascular-associated steps have not yet been fully connected within a single gastric-cancer study system.

Longitudinal spatial evidence for this category in gastric cancer remains limited. A small serial-biopsy treatment cohort has provided evidence of treatment-associated vascular normalization and changes in tumor–immune–vascular spatial relationships during ramucirumab plus pembrolizumab therapy [109]. However, independent paired cohorts, direct measurements of local drug exposure or functional perfusion, tracking of perivascular residual cells, and neighborhood-directed functional perturbation remain lacking. Accordingly, this niche is best viewed as a hypothesis-generating spatial unit associated with vascular-related migration, adhesion, and potential heterogeneity in drug delivery. Evidence linking this niche to treatment response is emerging but has not yet reached clinical validation.

### 2.5. Organ-Context-Dependent Adaptive Niche-Forming Process at Metastatic Sites

The metastatic-site adaptive process discussed in this section describes how disseminated gastric cancer cells re-establish interactions with local tissues after arrival at metastatic sites; it does not include the premetastatic niche, which exists before tumor-cell dissemination and arrival. Because organs differ substantially in resident cell populations, stromal architecture, vascular organization, and therapeutic exposure, this category should not be interpreted as a single uniform niche. Peritoneal metastasis currently represents the most developed gastric cancer-specific example, whereas lymph-node, brain, and other metastatic settings remain separate and less mature candidate subtypes. Shared functional processes across organs may include early survival, immune escape, local adhesion, vascular utilization, metabolic adaptation, and subsequent expansion. However, cellular composition, structural context, molecular programs, and validation criteria must be defined independently for each metastatic organ [110,111,112].

#### 2.5.1. Peritoneal Metastasis: Organ-Specific Reorganization of the Local Microenvironment

Peritoneal metastasis involves multiple interacting compartments, including the mesothelial surface, peritoneal stroma, peritoneal-cavity myeloid populations, CAFs, the ascitic environment, and local vasculature. Digital spatial analyses of primary gastric tumors, peritoneal metastases, and adjacent peritoneal tissues have shown that peritoneal metastases exhibit stromal, macrophage-related, and therapeutic-target features distinct from those observed in primary tumors and other metastatic sites, providing in situ spatial clues for local microenvironmental reorganization at peritoneal metastases. In the same study, evidence for peritoneal conditioning before the arrival of disseminated cells has been supported mainly by comparisons of early gastric cancer samples and humanized mouse models rather than by longitudinal human evidence [113]. Single-cell studies and treatment cohorts further suggest that the SPP1^+^ TAM–THBS2^+^ CAF stromal–myeloid axis is associated with the microenvironment of peritoneal metastasis and immune checkpoint therapy response, with functional support provided by C3–C3AR1 blockade experiments [114,115]. Free-floating cells or cellular states in ascites should not be considered equivalent to the spatial niche of a solid peritoneal implant and can be considered in situ spatial evidence only when linked to the location of the mesothelium, stroma, or a solid lesion.

#### 2.5.2. Lymph Nodes, Brain, and Other Metastatic Sites: Evidence Should Be Interpreted Separately

Gastric cancer cell states and immune compartments are reorganized in metastatic lymph nodes, and patient-matched spatial analyses of primary tumors and local metastatic lesions also show regional expression and immune-environment differences accompanying tumor spread [116,117]. These studies, however, primarily support interlesional heterogeneity and local tissue reorganization and are not sufficient to establish a unified lymph-node adaptive niche. Spatial transcriptomic analysis of gastric-cancer brain metastases has shown that angiogenesis and vessel co-option can coexist within the same lesion, accompanied by distinct T-cell, macrophage, and cytokine contexts [118].

Accordingly, this category should be evaluated using a common framework comprising (1) an organ-specific spatial context; (2) a neighborhood between gastric cancer cells and organ-resident components; (3) local survival, immune, vascular, or metabolic programs; and (4) colonization or expansion outputs, while the specific cellular composition and markers must be defined separately for each metastatic organ [111,113,116,117,118]. These data support spatial and molecular heterogeneity across metastatic organs but do not establish a uniform composition, universal marker set, or unified “resistance niche” applicable across all metastatic sites.

The defining elements, functional programs, associated clinical phenotypes, and representative evidence sources for each candidate spatial niche are summarized in Table 2.

### 2.6. Cross-Niche Comparison of Shared Functional Programs

Tissue-interface remodeling, failure of immune clearance, and stress adaptation–survival support are not three additional, independent niches, but cross-cutting functional axes used to compare the five spatial contexts. The invasive front is characterized by tissue-interface remodeling, with basement-membrane disruption and local infiltrative expansion as its outputs. Perivascular regions also involve interface proximity together with related adhesion, chemotaxis, and vascular-remodeling programs. At metastatic sites, the same interface-remodeling program may contribute to adhesion to organ surfaces, anchoring to the local matrix, and colonization.

Failure of immune clearance in the primary tumor may manifest as restricted CD8^+^ T-cell entry or post-infiltration functional dysfunction, whereas at metastatic sites it may manifest as organ-specific reorganization of immune compartments. Stress adaptation–survival support in poorly perfused regions is characterized mainly by tolerance to hypoxia, acidosis, and redox stress; in post-treatment lesions, it may be expressed as residual-cell survival, whereas in metastatic organs it may involve anoikis resistance and organ-specific metabolic adaptation. Thus, the same functional program can produce different malignant outputs depending on spatial context.

These three programs also help explain partial overlap among niches. For example, the invasive front may simultaneously exhibit hypoxia and immune exclusion, whereas peritoneal metastases may concurrently show stromal remodeling, myeloid immunosuppression, and metabolic stress.

## 3. Boundaries of Evidence Interpretation: Static Observations, Disease Heterogeneity, and Technical Limitations

### 3.1. Static Spatial Observations Are Not Equivalent to Dynamic Niche Evolution

Most current spatial studies of gastric cancer are based on single-time-point surgical specimens, cross-sectional comparisons among patients, noncontinuous sampling of primary and metastatic lesions, or analyses of spatial colocalization and clinical correlations. Such evidence can support pretreatment spatial heterogeneity and differences in biological states between lesions, but cannot directly establish continuous niche evolution over the course of disease. Differences between primary and metastatic lesions may also be influenced by multiple factors, including organ context, clonal selection, sampling methods, and prior treatment [110,117,123].

Dynamic interpretation should distinguish at least four scenarios: niches that pre-exist before treatment, niches selectively retained after treatment, niches induced or remodeled during treatment, and niches re-established after disseminated cells colonize a new organ. The first two can be distinguished by comparing pretreatment samples with residual lesions, whereas the latter two additionally require paired samples, time-series data, or functional models that preserve spatial organization. Accordingly, in the absence of longitudinal evidence, terms such as “formation,” “remodeling,” “transition,” and “continuous process” are used in this review only as process-based inferences or components of a conceptual framework.

### 3.2. Heterogeneity in Gastric Cancer Subtypes, Treatment Context, and Sampling

Gastric cancer does not represent a single spatial ecosystem. Intestinal and diffuse histological types, different Lauren classifications, microsatellite instability (MSI), Epstein–Barr virus (EBV) status, chromosomal instability and genomically stable subtypes, and HER2- or CLDN18.2-related molecular subgroups may differ in adhesive properties, stromal responses, and immune infiltration patterns [64,124,125,126,127]. For example, invasion-associated spatial reprogramming observed in diffuse-type gastric cancer cannot be directly generalized to all gastric cancers; similarly, neighborhood features identified in CLDN18.2-related multiplex-imaging cohorts and specific immunotherapy cohorts require independent validation across other molecular subgroups [64].

Treatment context likewise affects evidence interpretation. Untreated primary tumors, residual tissues after chemotherapy or targeted therapy, pretreatment immunotherapy biopsies, and post-progression samples represent distinct treatment contexts and temporal stages and should not be pooled without appropriate treatment annotation. Discordance in markers such as PD-L1 and HER2 between primary and metastatic lesions can support evidence of interlesional heterogeneity but cannot independently establish that the metastatic organ environment caused adaptive changes or acquired resistance [128,129].

Future clinical validation should evaluate whether spatial metrics provide incremental value beyond established clinicopathological and molecular variables, including TNM stage, Lauren classification, molecular subtype, PD-L1 CPS, HER2 status, and treatment-specific biomarkers, through appropriately powered cohorts and prespecified statistical models.

Sampling strategy strongly influences which spatial niches can be detected and which may remain undetected. A single two-dimensional section captures only a local plane of a tumor and cannot fully represent its three-dimensional spatial architecture. Endoscopic mucosal biopsies of gastric cancer primarily sample the superficial mucosa and submucosa and therefore may not adequately represent deep invasive regions or other internal spatial units. Results derived from limited regions of interest (ROIs) are influenced by ROI number, ROI area, and intratumoral heterogeneity. Moreover, when primary and metastatic lesions differ in sampling site, specimen type, tissue quantity, or fixation and processing procedures, their spatial and molecular features should not be assumed to be directly equivalent [117,130,131,132,133]. Consequently, failure to detect a particular niche in a sample does not indicate that the niche is absent from the patient, and multiregion sampling and three-dimensional reconstruction should be prioritized in future validation studies.

### 3.3. Evidentiary Capabilities of Different Spatial Technologies and Literature Classification

Dissociation-based single-cell sequencing is well suited for identifying cellular subtypes, cellular states, and potential communication relationships, but lacks direct in situ spatial adjacency information. Conventional IHC provides marker-expression information and limited spatial localization, but is less suited for robust definition of multicellular neighborhoods. Spot-level spatial transcriptomics preserves tissue location but may contain mixed signals from multiple cell types. Multiplex immunofluorescence/multiplex immunohistochemistry (mIF/mIHC) and digital pathology can support single-cell distance measurements and neighborhood analyses, although the results depend on segmentation algorithms, neighborhood definitions, and ROI selection. Spatial proteomics, spatial metabolomics, and high-resolution in situ transcriptomic technologies can enhance spatially resolved functional phenotyping, but still require integration with pathological architecture and functional validation experiments [17,18,19,20,21,25,26,27,28,134,135].

Accordingly, interpretations in the literature should be aligned with the actual capabilities of the study technology. Single-cell sequencing is used primarily to identify cell types and cellular states that may participate in biological processes, IHC can establish the tissue localization of marker expression, multiplex imaging or single-cell-resolution spatial technologies can characterize cellular composition and neighborhood relationships, and spatial transcriptomics or spatial proteomics can be used to infer candidate functional programs within local neighborhoods. Collectively, these approaches mainly support characterization of cellular composition, spatial associations, and functional hypotheses, whereas causal effects still require perturbation experiments, longitudinal studies, and models that preserve spatial architecture.

### 3.4. Operationalization and Recommended Reporting of Spatial-Niche Metrics

Spatial neighborhoods should be operationalized using prespecified and transparently reported spatial rules appropriate to the biological question, tissue architecture, and analytical platform. Depending on the study design, neighborhood relationships may be characterized using distance-based proximity, k-nearest-neighbor relationships, graph-based adjacency, structure-defined boundaries, or other explicitly specified spatial approaches. Because spatial resolution, tissue architecture, segmentation strategies, and biologically relevant scales vary substantially across platforms and niche types, the present framework does not prescribe a universal neighborhood radius, k value, ROI size, or numerical cutoff for reproducibility metrics. Instead, investigators should clearly report how spatial units and neighborhoods were defined, how ROIs or tissue regions were selected, the number and distribution of sampled regions, relevant tissue-region inclusion and exclusion criteria, segmentation and cell-phenotyping procedures, and major sources of technical variation. Platform-specific quality-control procedures should also be prespecified and transparently reported, including, where applicable, segmentation and phenotype-calling quality assessments, positive and negative assay controls, normalization and batch handling, scanner or platform effects, analysis-pipeline or model-version information, and predefined criteria for excluding technically inadequate regions or samples. These quality-control procedures should be tailored to the assay and intended use rather than replaced by a single universal cross-platform threshold.

Where multiple analytically reasonable parameter settings are possible, the robustness of resulting spatial metrics or niche assignments should, where feasible, be examined through sensitivity analyses across biologically plausible alternatives. Such analyses may include changes in neighborhood scale, ROI composition or sampling strategy, segmentation or phenotype-calling settings, and batch-adjustment procedures, as appropriate for the platform. Continuous spatial measures, including cell-density ratios, proximity scores, adjacency measures, and nearest-neighbor distances, should preferably be retained as continuous variables during model development rather than dichotomized using arbitrary universal thresholds. When categorical thresholds are required for prognostic or treatment-response applications, they should be derived in an appropriate development cohort, prespecified before external validation, and subsequently evaluated in independent cohorts. Where reproducibility is quantitatively evaluated, continuous spatial metrics may be assessed using the intraclass correlation coefficient (ICC) with 95% confidence intervals. Categorical framework assignments may be evaluated using Cohen’s κ, whereas ordered evidence categories may additionally be assessed using weighted κ. The selection of reproducibility thresholds should be prespecified according to the research question, spatial platform, and analytical workflow; no universal cutoff should be assumed across technologies or biological contexts. The purpose of such analyses is to determine whether the direction and interpretation of the spatial association are robust to reasonable analytical choices, rather than to establish one parameter setting that is universally applicable across technologies or niche types.

Accordingly, the present framework is intended to standardize terminology, reporting, and evidence interpretation rather than to impose platform-independent technical cutoffs. Supplementary Table S2 therefore provides a recommended reporting checklist for the operational identification and quantification of candidate spatial niches, whereas Supplementary Table S3 provides an illustrative application of the framework to representative published gastric cancer studies. These materials are intended to improve transparency and comparability rather than to constitute formal technical validation of a fixed classifier. Formal evaluation of test–retest reliability, inter-rater reproducibility, cross-platform concordance, external-cohort validation, and clinically acceptable performance thresholds should be undertaken in future validation studies once niche-specific definitions, analytical pipelines, and intended clinical uses have been sufficiently specified.

## 4. Spatial-Niche-Guided Clinical Translation: From Quantifiable Metrics to Validation Priorities

The translational value of spatial-niche research is not to replace TNM staging or molecular classification with new spatial labels, but rather to provide complementary information on local tissue architecture, cellular neighborhoods, and functional programs. A spatial feature should be further developed as a risk or treatment-response predictor only when it can be reproducibly quantified, replicated across platforms and cohorts, and demonstrated to provide incremental value beyond established clinical variables. Because the five framework categories differ in available evidence, sample composition, and validation maturity, their translational pathways should not be considered parallel or equivalent.

A relevant precedent is Immunoscore, which illustrates that spatially informed biomarkers require standardized analytical procedures, independent external validation, and clinical evaluation before translation into routine practice [136,137]. The development of Immunoscore from spatial characterization of immune-cell distribution to a clinically evaluated biomarker highlights the importance of analytical harmonization, multicenter validation, and prespecified clinical assessment. This experience provides a useful reference for candidate spatial niches in gastric cancer, which currently require further operational definition, quantitative standardization, and prospective evaluation before potential clinical implementation.

### 4.1. Patient Risk Stratification

Conventional risk stratification in gastric cancer relies mainly on TNM stage, pathological grade, Lauren classification, molecular subtype, and treatment-related biomarkers [124,125]. Spatial metrics may serve as complementary variables, but priority should be given to features with relatively high pathological reproducibility, clearly defined sampling requirements, and existing associations with clinical outcomes. Tumor budding, basement-membrane disruption, and interface-remodeling features at the invasive front have substantial pathological evidence and outcome associations and can therefore be prioritized for evaluation of incremental predictive value beyond existing staging and pathological variables. The intratumoral/stromal CD8 ratio and adjacency relationships associated with immune exclusion may be evaluated as immune-state stratification variables, whereas hypoxia- and perivascular-related metrics are more dependent on multiregion sampling and functional measurements.

Risk models should explicitly incorporate age, sex, TNM stage, pathological grade, Lauren/histological classification, molecular subtype, treatment exposure, sampling site, specimen type, and primary-versus-metastatic origin as appropriate. The number of patients and outcome events—not the number of cells or ROIs—should anchor sample-size planning. Multiple ROIs, sections, or lesions from one patient create a hierarchical data structure and should be handled using multilevel or clustered methods rather than being treated as independent observations. Future studies should test whether spatial metrics improve prediction of metastasis or recurrence beyond TNM stage, histology, and molecular classification, rather than constructing an independent spatial classification intended to replace existing systems [14,53,54,123,126,127,138].

### 4.2. Prediction of Therapeutic Efficacy: Distinguishing Specific Phenotypes of Treatment Resistance

Prediction of therapeutic efficacy must specify both treatment modality and temporal stage. The immune exclusion–immune evasion niche primarily corresponds to primary nonresponse or limited benefit from immune checkpoint inhibitors or combined immunotherapy and antiangiogenic treatment. The hypoxia–metabolic adaptation niche primarily corresponds to insufficient local drug exposure, reduced sensitivity to chemotherapy or targeted therapy, and post-treatment residual survival. The metastatic-site adaptive niche-forming process primarily reflects discordance in target expression and immune context between primary tumors and metastatic sites. At present, the perivascular supportive niche is used mainly to explore vascular-associated migration, spatial relationships with immune cells, and potential heterogeneity in drug delivery [68,87,90,117,139]; emerging serial-biopsy evidence suggests an association between vascular remodeling and treatment response [109], but independent validation is lacking.

Candidate metrics that can be prioritized for near-term validation include the pretreatment degree of CD8^+^ T-cell spatial exclusion, tumor–myeloid/CAF neighborhoods, hypoxic and metabolic programs in poorly perfused regions, and spatial differences in therapeutic targets in paired primary–metastatic samples. For treatment-response models, the comparator set should be therapy-specific: PD-L1 CPS and MSI/MMR for checkpoint-based therapy; HER2 status for HER2-directed therapy; CLDN18.2 status for CLDN18.2-directed therapy; and other validated biomarkers according to the regimen. Treatment line, prior therapy, biopsy timing, response criteria, and sampling site should be prespecified, because a spatial metric cannot demonstrate added value if these sources of heterogeneity are ignored [64,65,68,140,141,142,143].

### 4.3. Combination Interventions and Validation Priorities

Priority directions for testing include evaluating whether stromal or myeloid modulation can restore T-cell entry and effector function in immune-exclusion niches, whether improving perfusion or targeting metabolic stress can reduce residual survival in hypoxia–metabolic adaptation niches, and whether organ-specific stromal–myeloid relationships contribute to maintenance of peritoneal metastases and to response to immune checkpoint therapy in the peritoneal metastatic niche [144,145,146,147,148,149].

Clinical translation of a spatial niche requires a stepwise validation pathway involving the following: standardized operational definitions and locked quantification parameters; model development with internal validation; independent external-cohort and cross-platform replication; validation of causal or dynamic roles using spatially preserving models or paired pre- and post-treatment samples where feasible; and, ultimately, prospective clinical evaluation. Incremental value should be tested by adding the spatial metric to an established base model and reporting likelihood-ratio comparison, change in C-index or time-dependent AUC as appropriate, calibration, and decision-curve or net-benefit analysis, rather than relying solely on univariable associations. The clinically validated Immunoscore provides a useful methodological precedent for assay standardization, observer/centre reproducibility, locked scoring, cross-cohort replication, and evaluation beyond TNM, although it is not assumed to transfer directly to gastric-cancer niches [137]. Table 3 therefore distinguishes metrics that can be quantified in the near term from those that still require mechanistic or longitudinal validation [134,135,150].

Validation priorities should differ among framework categories. The invasive front should focus on perioperative metastatic risk; immune-exclusion and hypoxic niches should focus on functional validation related to treatment response; the perivascular niche should prioritize operational definition and local functional measurement; and metastatic-site adaptive processes should be studied separately by organ [151,152].

## 5. Conclusions and Perspectives

The value of a spatial-niche framework is not in renaming existing spatial phenomena, but rather in establishing an evidence framework linking tissue context, neighborhoods characterized using prespecified and transparently reported spatial rules, and local functional programs with malignant phenotypes or clinical outcomes. At present, gastric cancer lacks spatial-niche metrics that have undergone sufficient clinical validation for routine application.

The next stage of research should prioritize three questions: whether candidate-niche assignments and spatial metrics can be reproducibly identified across patients, subtypes, sampling methods, and platforms; whether key neighborhoods have functional roles in metastasis or specific treatment-failure phenotypes; and whether pre-existing, selectively retained, and treatment-induced or treatment-remodeled states can be distinguished using longitudinal samples. Only after these questions are addressed can spatial niches move beyond descriptive tissue maps toward operational variables for gastric cancer metastasis research, therapeutic-response prediction, and combination-intervention design.

## Figures and Tables

**Table 1:** Four-Stage Evidence Framework and Minimum Criteria for Candidate Spatial Niches.

Evidence Stage	Minimum Criteria	Conclusions Supported	Conclusions Not Supported by This Evidence Alone
Descriptive spatial evidence	Spatial patterns, spatial domains, and/or cellular neighborhoods show nonuniform distribution, regional boundaries, or quantifiable adjacency	Spatial heterogeneity and organized local spatial structure are present	The local structure may have a functional role or already constitutes a niche
Candidate-niche stage	Defined spatial context + a neighborhood characterized using a prespecified and transparently reported spatial rule + a concordant local functional program + association with a malignant phenotype or clinical outcome; robustness to reasonable analytical choices assessed where applicable	The local unit can be compared and tested as a functional-niche hypothesis	Necessity, sufficiency, continuous evolution, or independent clinical predictive value
Functional-support stage	On the basis of a candidate niche, spatial perturbation, spatially preserving models, in vivo tracking, or paired/longitudinal evidence is available	The functional relationship between the key neighborhood and the corresponding phenotype is supported by perturbational, longitudinal, or model-based evidence	Generalizability without cross-cohort replication and clinical predictive performance
Clinical-validation support stage	Independent replication across cohorts and, where relevant, platforms, with consistent associations and incremental value demonstrated in multivariable or prospective studies	The feature can be further developed as a variable for risk stratification or prediction of therapeutic efficacy	Direct replacement of existing clinical classifications or use as a sole determinant of treatment

Note: Spatial patterns, spatial domains, and cellular neighborhoods are complementary forms of descriptive spatial evidence rather than sequential evidence stages. The four-stage framework distinguishes evidence from description and candidate designation through functional support to clinical validation. It does not prescribe universal values for neighborhood scale, ROI size, or reproducibility thresholds; study-specific spatial rules should instead be prespecified, transparently reported, and examined for robustness to reasonable analytical choices where applicable. Detailed considerations regarding spatial metric quantification, threshold selection, reproducibility assessment, and validation strategies are discussed in Section 3.4.

**Table 2:** Spatial Scale, Niche-Defining Criteria, Functional Outputs, Associated Clinical Phenotypes, and Representative Evidence Sources for the Five Spatial Framework Categories.

Spatial Framework Category	Spatial Scale and Stage	Candidate-Defining Elements	Dominant Functional Programs and Associated Outputs	Corresponding Malignant Clinical Behavior or Treatment-Failure Phenotype	Representative Evidence Sources
Invasive front–tumor–stroma interface niche	Deepest invasive front of the primary tumor; metastatic-initiation stage	Tumor budding/dispersed cells + basement-membrane or ECM disruption + supportive stromal/myeloid neighborhood + interface-remodeling program	Characterized by tissue-interface remodeling; associated outputs include local infiltrative expansion, lymphovascular invasion, and early dissemination	Primarily associated with perioperative metastatic/recurrence risk; no clear evidence currently links this niche to acquired resistance to a specific drug	Direct gastric-cancer evidence: refs. [51,52,57] Cross-cancer mechanistic support: ref. [58]
Immune exclusion–immune evasion niche	Primary-tumor margin, stroma, and tumor nests; pretreatment immune state	Entry restriction: CD8 marginal retention/increased distance + CAF/ECM barrier; functional dysfunction: intratumoral CD8 + low effector programs/suppressive networks	Associated with impaired immune clearance; associated outputs include immune escape and selective tumor-cell survival	Primary nonresponse/limited benefit from immune-checkpoint or immunotherapy–antiangiogenic treatment; insufficient evidence for acquired resistance. Relatively stronger treatment-response-related evidence is available, but complete clinical validation remains lacking.	Direct gastric-cancer evidence: refs. [14,61,64,65,68] Cross-cancer mechanistic support: ref. [119]
Hypoxia–metabolic adaptation niche	Poorly perfused, vessel-distant, or necrotic-border regions of the primary tumor; stress-adaptation stage	Low-perfusion context + hypoxia/glycolysis/acidification programs + viable tumor cells + stress-adaptation output	Characterized by stress adaptation–survival support, with associated immunosuppressive features	Reduced sensitivity to chemotherapy/targeted therapy, insufficient drug exposure, and residual survival; not equivalent to acquired resistance	Direct gastric-cancer evidence: refs. [73,89,90,120] Cross-cancer mechanistic support: ref. [121]
Perivascular supportive niche	Tumor–vascular interface in the primary tumor; vascular-associated migration and perfusion context	Clear and quantifiable tumor–vascular proximity + endothelial/pericyte structures + chemotactic, adhesive, or vascular-remodeling programs	Characterized by tumor–vascular interface organization, with associated adhesion, chemotactic, and vascular-remodeling programs	Potential uneven drug delivery or local exposure differences and treatment-associated vascular remodeling can be investigated; a defined resistance or residual-cell sanctuary function remains unvalidated.	Direct gastric-cancer evidence: refs. [68,109] Cross-cancer mechanistic support: ref. [122]
Organ-context-dependent adaptive niche-forming process at metastatic sites (peritoneum best supported)	Peritoneum; lymph nodes, brain, and other sites analyzed separately; colonization and expansion stage	Organ-specific context + gastric cancer cell–resident component neighborhood + local survival/immune/vascular/metabolic programs + colonization output	All three functional programs may participate; outputs vary by organ and include adhesion, immune evasion, vascular utilization, and expansion	Differences in efficacy/targets between primary and metastatic lesions and metastatic-site-specific low response; not a uniform form of acquired resistance	Direct gastric-cancer evidence: peritoneum [113,114,115]; lymph node [111,116,117]; brain [118] Nonperitoneal sites remain unresolved organ-specific candidate subtypes

Note: All five categories are compared using the same minimum criteria chain, but they differ in spatial scale, stage of formation, and current evidence base. The table lists representative gastric-cancer evidence and cross-cancer mechanistic support rather than an exhaustive summary of the literature; evidence from different sources should be interpreted according to the methodological capabilities and limitations of the corresponding study designs. The “associated clinical phenotypes” shown refer only to the specific settings addressed by existing studies and do not imply that the five categories have equivalent evidence maturity or clinical translational status.

**Table 3:** Near-Term Quantifiable Metrics and Validation Priorities for the Five Spatial Framework Categories.

Spatial Framework Category	Near-Term Quantifiable Metrics	Corresponding Clinical Question	Interpretation And Research Use Supported by Current Evidence	Key Next Validation
Invasive front–tumor–stroma interface niche	Tumor-bud density, deepest invasive front, collagen IV continuity, FAP/CD68 neighborhoods, LAMC2/MMP7 programs	Perioperative metastatic and recurrence risk	Candidate pathological–spatial risk variable; insufficient evidence for prediction of efficacy of specific drugs	Multiregion replication; determine whether multicellular neighborhoods provide incremental value independent of tumor budding
Immune exclusion–immune evasion niche (TLS as favorable comparator)	Continuous intratumoral/stromal CD8 ratio, nearest-neighbor distance, CAF/ECM barrier, intratumoral effector programs, myeloid/Treg neighborhoods, and TLS-referenced organization	Primary nonresponse or limited benefit from immunotherapy	Multiplex imaging, spatial-organization metrics, and treatment-response cohorts support candidate predictive value; entry restriction should be distinguished from post-infiltration dysfunction	Derive and lock any clinically used threshold in a development cohort; assess robustness across reasonable analytical choices where feasible; obtain paired pre/post-treatment samples and functional perturbation; validate in independent immunotherapy cohorts; compare with established biomarkers and favorable TLS reference states.
Hypoxia–metabolic adaptation niche	Vessel distance/functional perfusion, CAIX/GLUT1, LDHA/MCT4, viable regions at necrotic borders, and local immune state	Reduced sensitivity to chemotherapy/targeted therapy, insufficient drug exposure, and residual survival	Can be investigated as a hypothesis for reduced sensitivity and residual-disease risk; acquired resistance still requires longitudinal evidence	Simultaneous measurement of perfusion, drug concentration, and residual cells; longitudinal tracking of post-treatment changes
Perivascular supportive niche	Tumor–vascular distance, endothelial/pericyte coverage, and local chemotactic/adhesive programs	Vascular-associated migration, treatment-associated vascular remodeling, and potential heterogeneity in drug delivery	Can be used to explore vascular-associated migration, vascular/immune spatial remodeling during therapy, and drug-delivery heterogeneity; resistance or sanctuary function remains unvalidated.	Independent serial/paired treatment cohorts; direct local drug-exposure or functional-perfusion measurements; perivascular residual-cell tracking; neighborhood-directed perturbation.
Organ-context-dependent adaptive niche-forming process at metastatic sites	Organ-specific quantification of tumor–resident-cell neighborhoods, stromal/immune/vascular-utilization programs, and target expression	Metastatic-site-specific colonization, therapeutic efficacy, and target differences	Peritoneal disease has digital spatial, single-cell, and treatment-cohort evidence; lymph nodes, brain, and other organs require separate definition and validation, with no uniform marker set	Organ-stratified cohorts, paired primary–metastatic samples, multiregion sampling, and associations with treatment response

Note: The metrics listed are near-term quantifiable research variables and validation priorities proposed on the basis of current evidence to guide future study design. ‘Near-term quantifiable’ does not mean that a metric already demonstrates stable clinical predictive performance, and the listed validation priorities do not represent an evidence hierarchy or a ranking of clinical recommendations across the different niches. Continuous metrics should be reported continuously and, when converted into categories, should use prespecified or development-cohort-derived thresholds that are locked before independent validation.

## Data Availability

All study-level evidence extraction and framework classifications generated for this review are provided in Supplementary Table S1 and the associated Search Log. No new primary patient-level or experimental data were generated.

## Supplementary Materials

The following supporting information can be downloaded at https://www.mdpi.com/article/10.3390/cells15181676/s1, Table S1, a 66-entry study-level evidence map with framework assignment, retrieval provenance, evidence-stage grading, and evidence boundaries; Search Log, formal PubMed/Web of Science rerun strings and counts, eligibility boundaries, deduplication audit, screening transparency, and included-study provenance; Table S2, recommended reporting checklist for the operational identification and quantification of candidate spatial niches; Table S3, illustrative application of the proposed framework to representative published gastric cancer studies; and Table S4, two-rater illustrative framework application and agreement assessment.
